# A novel homozygous missense substitution p.Thr313Ile in the *PDE6B* gene underlies autosomal recessive retinitis pigmentosa in a consanguineous Pakistani family

**DOI:** 10.1186/s12886-023-02845-0

**Published:** 2023-03-23

**Authors:** Nobia Aziz, Mukhtar Ullah, Abdur Rashid, Zubair Hussain, Khadim Shah, Azeem Awan, Muhammad Khan, Inam Ullah, Atta Ur Rehman

**Affiliations:** 1 Department of Biotechnology and Genetic Engineering, Faculty of Biological and Health Sciences, Hazara University, Mansehra, Pakistan; 2 Institute of Molecular and Clinical Ophthalmology Basel, University of Basel, Basel, Switzerland; 3 Department of Ophthalmology, University of Basel, Basel, Switzerland; 4 Department of Higher Education Archives and Libraries Peshawar, Government of Khyber Pakhtunkhwa, Peshawar, Pakistan; 5 Department of Biotechnology, COMSATS University Islamabad, Abbottabad Campus, Abbottabad, Pakistan; 6 LRBT Secondary Eye Hospital, Reerah Galla, Balakot Road, Mansehra, Khyber Pakhtunkhwa Pakistan; 7 Department of Zoology, Faculty of Biological and Health Sciences, Hazara University, Mansehra, Pakistan

**Keywords:** Inherited retinal disorders, Homozygous, Autozygosity, Retinitis pigmentosa, *PDE6B*, Pakistan

## Abstract

**Background:**

Retinitis pigmentosa (RP) is one of the most frequent hereditary retinal diseases that often starts with night blindness and eventually leads to legal blindness. Our study aimed to identify the underlying genetic cause of autosomal recessive retinitis pigmentosa (arRP) in a consanguineous Pakistani family.

**Methods:**

Following a detailed ophthalmological examination of the patients by an ophthalmologist, whole-exome sequencing was performed on the proband’s DNA to delineate the genetic cause of RP in the family**.** In-depth computational methods, in-silico analysis, and familial co-segregation study were performed for variant detection and validation.

**Results:**

We studied an inbred Pakistani family with two siblings affected by retinitis pigmentosa. The proband, a 32 years old female, was clinically diagnosed with RP at the age of 6 years. A classical night blindness symptom was reported in the proband since her early childhood. OCT report showed a major reduction in the outer nuclear layer and the ellipsoid zone width, leading to the progression of the disease. Exome sequencing revealed a novel homozygous missense mutation (c.938C > T;p.Thr313Ile) in exon 12 of the *PDE6B* gene. The mutation p.Thr313Ile co-segregated with RP phenotype in the family. The altered residue (p.Thr313) was super conserved evolutionarily across different vertebrate species, and all available in silico tools classified the mutation as highly pathogenic.

**Conclusion:**

We present a novel homozygous pathogenic mutation in the *PDE6B* gene as the underlying cause of arRP in a consanguineous Pakistani family. Our findings highlight the importance of missense mutations in the *PDE6B* gene and expand the known mutational repertoire of *PDE6B*-related RP.

**Supplementary Information:**

The online version contains supplementary material available at 10.1186/s12886-023-02845-0.

## Introduction

Retinitis pigmentosa (RP) is a group of hereditary retinal disorders (HRDs) that affect nearly 1 in 4000 people worldwide [1]. However, the prevalence of RP varies across world populations, and the condition being highly frequent in the South Asian countries (1/930 in South India, 1/1000 in Northern China [2, 3]. RP typically appears with a loss of peripheral vision causing visual field constriction (tunnel vision) followed by central vision loss, and eventually leads to complete blindness though visual acuity may remain unaffected during the whole course of the disease. These symptoms are mainly due to progressive degeneration of rods and cones photoreceptors cells of the retina [4, 5]. As of today, mutations in at least 89 genes have been reported in RP patients following all modes of Mendelian inheritance, including autosomal dominant (AD), autosomal recessive (AR), X-linked, and mitochondrial or digenic inheritance (RetNet: https:/sph.uth.edu; accessed on 18 February 2022).

Phosphodiesterase 6 (PDE6) remains integral to the vertebrate phototransduction pathway because it regulates the cytoplasmic level of cyclic guanosine monophosphate (cGMP) in the photoreceptors [6]. It comprises a total of four subunits, including, a catalytic alpha-subunit *PDE6A* (MIM # 613810), a catalytic beta subunit *PDE6B* (MIM # 163500, 613801), and two inhibitory gamma-subunits *PDE6G* (MIM # 613582) [7, 8]. The enzyme hydrolyzes the intracellular cytoplasmic cGMP level [9]. Consequently, low level of cGMP leads to the closure of ion channels and therefore membrane hyperpolarization [10]. The gene encoding the beta subunit of PDE6 (*PDE6B*) was one of the foremost genes known to cause retinal degeneration in mice, dogs, and humans [7, 8]. *PDE6B* harbors a 45-Mb region on human chromosome 4p16.3 and is composed of 22 exons, which give rise to several splice isoforms, ranging in length from 2.7 to 3.4 Kb [11]. Loss-of-function (LoF) mutations in the *PDE6B* gene lead to dysfunction of the PDE6 holoenzyme and an accumulation of cGMP and Ca^2+^ in the rod photoreceptor cells. Accumulations of cGMP and Ca^2+^ lead to degeneration of rods followed by cone cells through apoptosis consequently leading to blindness. Recessive bi-allelic mutations in *PDE6B* are a common cause of autosomal recessive RP (arRP) in various populations [11, 12]. Nevertheless, heterozygous dominant mutations in the *PDE6B* gene cause AD congenital stationary night blindness [13]. Among patients with arRP, ~ 5–8% cases are known to have defects in rod-specific cyclic guanosine monophosphate (cGMP) phosphodiesterase 6β subunits (*PDE6B*) [14]. Similarly, mutations in the *PDE6B* homolog (NM_000283.3) have been known to cause rod and cone degeneration in animal models [6, 15].

Molecular diagnosis of RP in clinical practice has been greatly facilitated, thanks to recent advances in next-generation sequencing (NGS) technologies, notably, whole-exome sequencing (WES) and whole-genome sequencing (WGS) [16]. Today, ophthalmic examinations coupled with NGS are considered as the most suitable technique for the molecular diagnosis of RP [5, 8]. Treatment options for *PDE6B*-related RP are currently not available; however, proof-of-concept studies with sub-retinal gene therapy showed positive effects in mice and dogs when treated at a very early time during postnatal development of the retina [17, 18]. A clinical trial on the safety and efficacy of gene therapy in human patients with RP caused by bi-allelic mutations in the *PDE6B* gene is currently ongoing (ClinicalTrials.gov Identifier: NCT03328130). Similarly, transplantation of chemically induced photoreceptor-like cells (CiPCs) into the sub-retinal space of *rd1* mice, which were homozygous mutants for *Pde6b*, showed a partial restoration of the pupil reflex and visual function [19]. In this study, we document clinical and molecular findings in a consanguineous Pakistani family segregating *PDE6B*-related RP.

## Materials and method

### Ethics statement and clinical investigation

This study was approved by the bioethical committee of Hazara University, Mansehra, Pakistan (Approval No. F.NO:185/HU/Zool/2021/182), and was conducted in accordance with the standards of the Declaration of Helsinki and according to the ARVO statement on the use of human subjects in medical research. Written informed consent was obtained from the parents of the patients. Clinical and demographic information was recorded on a pre-designed questionnaire through face-to-face interviews with the patients and the pedigree of the family was drawn electronically using the pedigree chart designer tool (CeGaT, Tubingen, Germany). Patients were clinically examined by an ophthalmologist at the Layton Rehmat Ullah Benevolent Trust (LRBT) Hospital, Mansehra, Pakistan. Ophthalmic examination included measurement of best-corrected visual acuity (BCVA) using a Snellen chart, slit-lamp biomicroscopy, and ophthalmoscopy, fundus examination after pupillary dilation, fundus photography, spectral domain optical coherence tomography (SD-OCT).

### Genetic analysis

Following collection of saliva samples from patients as well as available healthy members of the family in a saliva self-collection kit (DNA Genotek, Ottawa, Canada), genomic DNA was extracted from saliva samples using a standardized protocol as mentioned in the prepIT-L2P manual (DNA Genotek, Ottawa, ON, Canada). Next quantitative and qualitative assessment of the proband’s DNA was made, and ~ 2.0 μg of genomic DNA was sent to Novogene Co. Ltd (Hong Kong, China) for whole-exome sequencing (WES) analysis. Protocols and platforms used for sequencing have been previously published [20, 21]. Exome data were analyzed using the *in-house* computational pipeline. Details of filtering steps and variant prioritization strategy were described previously [21]. All short-listed variants were classified based on their molecular profile (nonsense, frameshift, missense, and splice sites). The nature of pathogenicity of all missense variants was checked using numerous online tools such as, but not limited to, Sorting Intolerant from Tolerant (SIFT), Polyphen-2, Mutation Taster, Provean and CADD, etc. Genome-wide autozygosity mapping was performed using AutoMap [22]. PCR-based amplification of the target DNA sequence carrying the potentially pathogenic variant(s) was done using standard PCR conditions/cycles, and by employing a pair of target-specific primers (Forward primer: 5’-TACCAAGGGCAGCACTCAA-3’ and Reverse primer: 5’-CACAGTGCTGGAGTACGGG-3’). PCR products were Sanger sequenced bi-directionally using a commercial facility. Lastly, causality of the potentially pathogenic variant(s) was confirmed by performing a strict genotype–phenotype co-segregation study within the available members of the family.

### Amino acid conservation

To examine the evolutionary conservation of the altered (p.Thr313) and adjacent residues, PDE6B protein sequences of different vertebrate species including Human (*H. Sapiens*, NP_001138764), Chimpanzee (*P. troglodytes* XP-024211748.1), Cattle (*B. taurus,* NP-776843.1), Rat (*R. norvegicus,* NP-001099494.1), Rhesus monkey (*M. mulatta,* XP-028704029.1), Chicken (*G. Gallus*, NP-001305369.1), Cat (*F. cactus,* XP-019685437.1), Horse (*E. caballus,* XP-023494350.1), Dog (*C. lupus*, NP-001002934.2), Zebrafish (*D. rerio* XP-685002.1), Woodpecker (*D. Pubescens*, XP-009907072.1), and Mouse (*M. Musculus*, NP-032832.2) were downloaded in fasta format from UniProtKB/Swiss-prot database (http://www.uniprot.org/). Fasta sequences were aligned using clustal omega multiple sequence alignment package (https://www.ebi.ac.uk/Tools/msa/clustalo/).

### Three-dimensional (3D) modeling of protein structure

Swiss Model was used to create homology models for wild (PDE6B and PDE6A) and mutant (p.Thr313Ile_PDE6B) proteins and energy was minimized by using UCSF Chimera 1.1 [23] with the default setting, and the models were evaluated using verify 3D [24], ERRAT [24, 25] and ProCheck [26]. The Ligand cGMP was retrieved from PubChem (135398570) [27]. The ligand was converted to 3D and was energy minimized using Chem 3D version 19.0.0.22 (PerkinElmer, Waltham, MA, USA). Active site residues were predicted using a 3D ligand Site [28]. MOE [29] was used for docking of wild-type and mutant PDE6B protein with cGMP ligand and PDE6A. Interaction between wild and mutated PDE6B with cGMP and PDE6A were analyzed by PyMol [The PyMOL Molecular Graphics System, Version 2.4 Schrödinger, LLC], while wild-type and mutated PDE6B were docked with PDE6A using CLUSPRO 2.0 [30].

## Results

### Clinical data

**Fig. 1:**
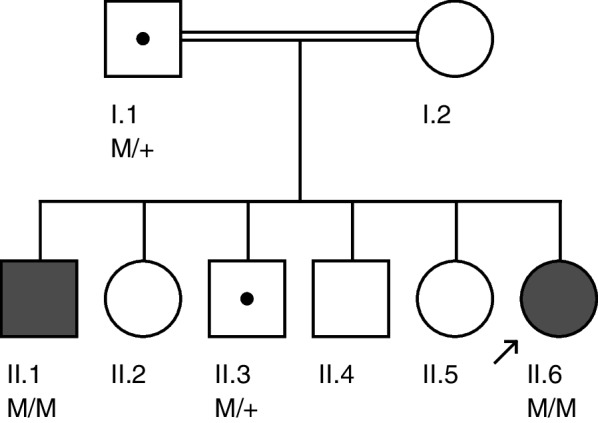
Pedigree of the family. Proband (II.6) in the pedigree is shown by an arrow. Dark-filled symbols represent affected individuals (II.1, II.6) while healthy individuals are shown as unfilled symbols. Symbols with a central dot indicate known heterozygote carriers

**Fig. 2:**
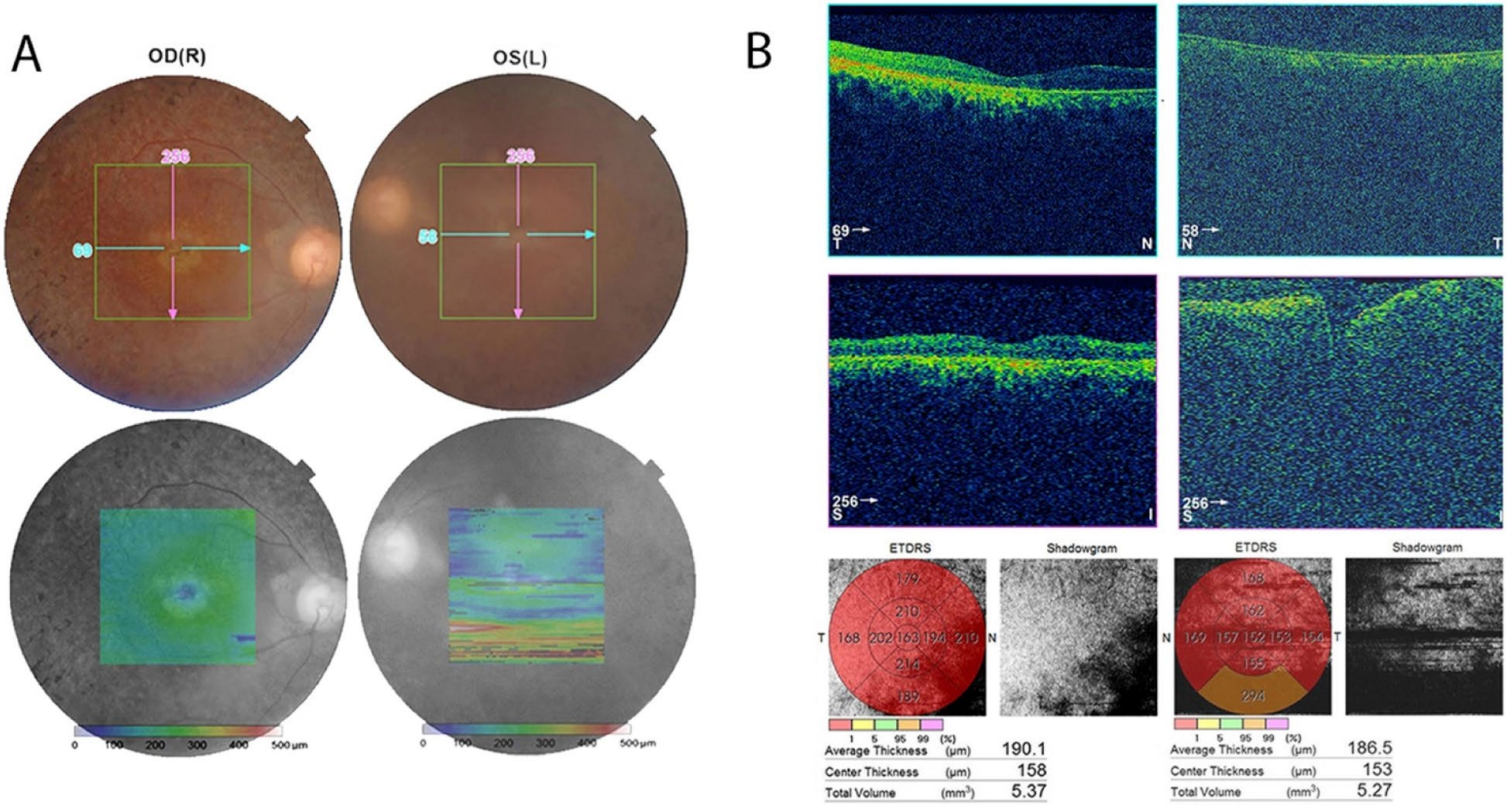
Fundus photograph and OCT images of the proband. **A** Colour fundus photographs of the proband revealed widespread macular atrophy, narrow vessels, optic disc pallor, and early pigmentary bone spicules. **B** OCT of the proband showing significantly reduced retinal thickness, and inner segment/outer segment (IS/OS) are not visualized in some parts of the retina

The pedigree of the family is shown in Fig. 1. The proband (II.6) is currently a 32-years old female living in Abbottabad district in northern Pakistan. She is the child of unaffected parents (I.1 and I.2) who married consanguineously (parents are first-cousins). The proband has five siblings, all alive, including three brothers and two sisters. Of all the siblings, only one brother (II.1) of the proband has RP while the remaining siblings were all healthy. The proband was suffering from night blindness at the age of 6 years and was diagnosed with RP by an ophthalmologist. She was aware of dark adaptation problems since the age of approximately 5 years. During her last examination in 2020, at age 31 years, the patient’s best-corrected visual acuity was greatly damaged, as both eyes were sensitive only to light perception. As shown in Fig. 2A, the fundus photograph of the proband showed arteriolar attenuation, waxy disc pallor, and mid-periphery bony spicules. There were also associated maculopathy with altered foveal reflex. Red free image with a superimposed false color-coded macular thickness map showed reduced macular thickness, indicating foveal atrophy. There were, however, no evidence of posterior vitreous detachment or abnormal vitreous attachment. The inner retinal layers (Retinal Nerve-fibre layer and Ganglion cell layer) appear grossly intact. There seems no intra retinal hypo/hyper reflective areas in the middle layers of retina namely Inner Plexiform layer, Inner nuclear layer, Outer Plexiform Layer and Outer Nuclear layer. The underline Retinal pigment epithelium shows degenerative changes. The corresponding map of age-matched normal is showing diffuse thinning in the foveal region. There is reduced average thickness, reduced central thickness and reduced total volume of 190 um, 158um and 5.37mm3 respectively (Fig. 2B).

### Genetic findings

**Fig. 3:**
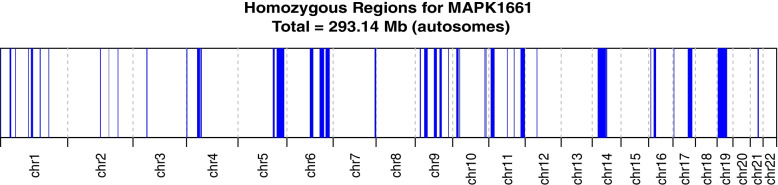
Homozygosity mapping showing genome-wide autozygous interval as vertical blue peaks along all the autosomes (1–22 chromosomes) shown on horizontal axis. The red arrow indicates an autozygous interval (~ 2.36 Mb) harboring *PDE6B* gene in which mutation was uncovered. Total autozygosity (293.14 Mb) is shown on top of the figure

Whole-exome sequencing in proband identified a homozygous missense change (NM_001145292.2:c.938C > T; p.Thr313Ile) in the *PDE6B* gene (NM_001145292.2). Interestingly, genome-wide homozygosity mapping of WES data localized the *PDE6B* gene inside an autozygous interval spanning ~ 2.36 Mb; however, total genomic autozygosity in the proband was found to be 293.14 Mb (Fig. 3). Collectively, these data corroborate pedigree-based consanguinity in the studied family.

**Fig. 4:**
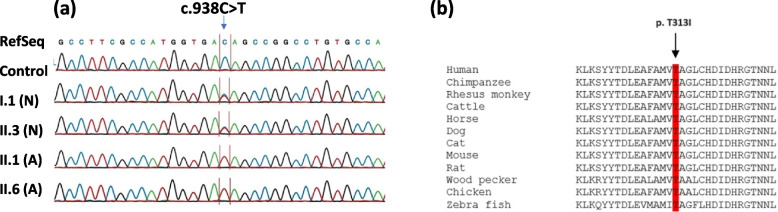
Sanger sequencing results and evolutionary profiling of the altered residue. **a** Chromatograms of the control sample were compared with available members of the studied family. As highlighted in a red box, c.938C > T sequence alteration can easily be seen in a homozygous state (single-peak) in both patients (II.1 & II.6) while proband’s father (I.1) and a healthy sibling (II.3) are carrying the same mutation in a heterozygous state (indicated by double-peaks). **b** Multiple sequence alignment showing p.T313I change inside a conserved region of the PDE6B protein (red highlighted). RefSeq: Reference Sequence, N: normal, A: affected with RP

Upon familial co-segregation study using DNA of the available members of the family, c.938C > T variation showed strict genotype–phenotype correlation. As shown in chromatograms in Fig. 4a, the mutation (c.938C > T) was present in a homozygous state in both patients (II.1, II.6) while proband’s father (I.1) and a healthy brother (II.3) were both heterozygote carrier for the same sequence alteration. This unique sequence variation was not detected in our internal control cohort which contains whole-exome and genome sequencing data from more than 500 unrelated individuals. However, c.938C > T alteration was detected heterozygously only once in the genome aggregation database (gnomAD; http://gnomad.broadinstitute.org/), which contains sequencing data from over 141,000 unrelated individuals. In-silico analysis of the variant using existing online tools classified the variant as deleterious. Similarly, multiple sequence alignment of the altered residue revealed a high degree of evolutionary conservation across species (Fig. 4b).

**Fig. 5:**
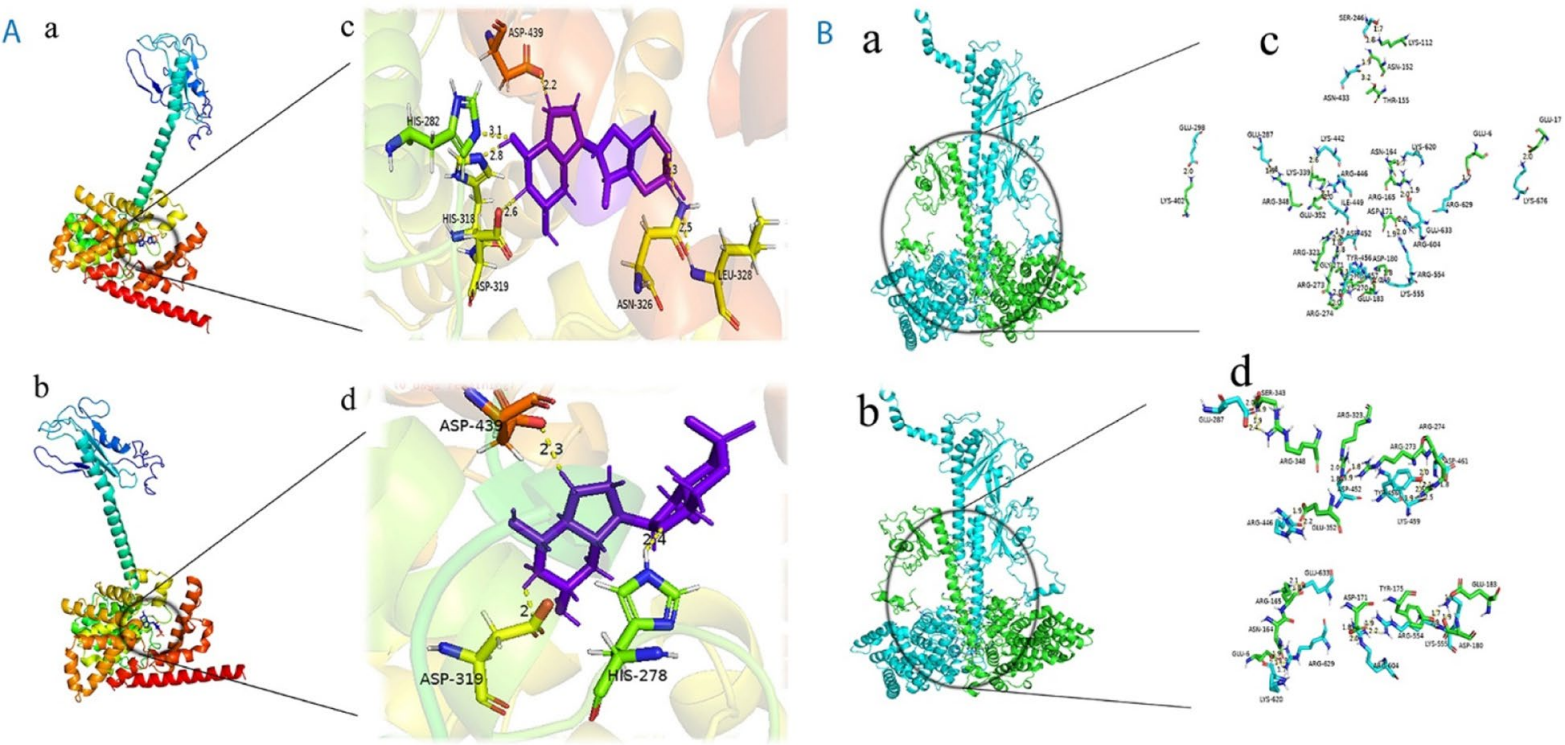
**A** The interaction of wild-type PDE6B and mutant type PDE6B with cGMP is shown. Surface representation of cGMP interactions with (a) wild type PDE6B and (b) mutant type PDE6B. 3D representation of cGMP (in purple) interactions with (c) wild type PDE6B and (d) mutant type PDE6B. **B** Depicts the interactions of wild-type PDE6B and mutant type PDE6B with wild type PDE6A. Surface representation of interactions between (a) wild type PDE6B (in green) with PDE6A (in cyan), and (b) mutant type PDE6B (in green) and PDE6A (in cyan). 3D interactions of PDE6A with (c) wild type PDE6B and (d) mutant type PDE6B

Docking scores for wild and mutated protein with cGMP were -16.9951 and -11.0597, which shows destabilization upon mutation (Fig. 5A). Docking results showed that the wild PDE6B interacts with cGMP through His279, Asp319, and Asp439 residues, while the mutant type PDE6B interacts with cGMP through His282, His318, Asp319, Asp326, Leu328 (Fig. 5A). Furthermore, wild-type and mutant PDE6B interacted with its heterodimer PDE6A via distinct hydrogen bonds and residues (Fig. 5B). Native and aberrant interactions of wild-type and mutated *PDE6B* with *PDE6A* are shown in supplementary data (Table S1).

## Discussion

Here, we report a homozygous missense substitution (c.938C > T; p. Thr313Ile) in the *PDE6B* gene as the underlying cause of arRP in a consanguineous northern Pakistani family. Two affected siblings in the family appeared to have nyctalopia as an early symptom, later progressing towards the classical RP phenotype. The mutation was identified by performing whole-exome sequencing analysis in the proband while its pathogenicity was confirmed by achieving several lines of evidence supporting the causality of our sequence variation. For instance, c.938C > T sequence alteration was never reported on any major public databases of human genome variation (HGMD, ClinVar, gnomAD, etc.), the altered residue (p.Thr313Ile) was super conserved evolutionarily across different vertebrate species, and nearly all in-silico pathogenicity predictors have classified the sequence variation as ‘pathogenic’. Upon segregation analysis, the mutation showed a strict genotype–phenotype correlation in the family such that the mutation was present in a homozygous state in both patients; however, parents and a sibling were heterozygote carriers for the mutation and thus remained clinically unaffected. As presumed in our pedigree-based analysis, our findings supported an autosomal recessive inheritance pattern of the disease which further corroborates several previous studies that implicated *PDE6B* mutations in arRP [31–33]. Finally, our 3D modeling/docking results showed an overall destabilization of the protein upon mutation leading to the loss of usual PDE6B interactions with its ligand cGMP and its heterodimer PDE6A while simultaneously creating aberrant interactions with its ligands/heterodimer via distinct hydrogen bonds and residues. These aberrant interactions of the mutant PDE6B with cGMP and PDE6A are suggestive of PDE6B dysfunction, possibly explaining the visual impairment phenotype in our patients.

Pathogenic mutations in the *PDE6B* gene are known to cause autosomal dominant congenital stationary night blindness (adCSNB) or autosomal recessive retinitis pigmentosa (arRP); however, similar mutations in *PDE6A* are known to cause only autosomal recessive retinitis pigmentosa (arRP) [34]. Nevertheless, the phenotypic analysis revealed no substantial differences between the two groups except for night blindness as a symptom that was noted to be more prevalent in the PDE6A-linked RP than in the *PDE6B* group [31, 35].

Thus far, over 200 pathogenic mutations in the *PDE6B* gene, largely missense/nonsense mutations (*n* = 132), have been reported on the HGMD database (Professional version 2021.4; last accessed March 2022). Mutations in *PDE6A* and *PDE6B* genes are not uncommon in Pakistan [36–38]. For example, around 2–3% of the total genetic load of arRP is attributable to pathogenic sequence variations in the PDE6 genes (*PDE6A* and PDE6B) [39]. Specifically, at least six distinct pathogenic mutations in the *PDE6B* gene are so far known in Pakistani families, including a splicing defect (c.1722 + 1G > A:p.?) [39], two missense mutations (c.1160C > T:p.Pro387Leu; c.1655G > A:p.Arg552Gln) [22], and three frameshift deletions (c.427del:p.Ala143LeufsTer7; c.243delG:p.Arg82AlafsTer68; c.12_15delTGAG:p.Ser4ArgfsTer23) [21, 40]. With the addition of our mutation into the literature, our family constitutes the 7^th^ *PDE6B*-linked RP family in Pakistan. In populations, such as Pakistani society, where consanguinity and community endogamy are commonplace, inherited disorders, in most cases, follow an autosomal recessive pattern of inheritance, and the underlying genetic perturbations are presumed to be present in a homozygous state due to its bi-parental inheritance [41]. To uncover such homozygous pathogenic mutations in consanguineous families, exome- or genome-sequencing coupled with autozygosity mapping remains a gold standard approach [39]. Following the same path, we are constantly uncovering the underlying genetic causes of several Mendelian disorders in Pakistani families [21, 42–44], thus suggesting the same approach to future researchers or clinicians dealing with monogenic families from inbred populations daily.

## Conclusion

In summary, we report a homozygous pathogenic missense mutation (p.Thr313Ile) in the *PDE6B* gene as the underlying cause of arRP in a consanguineous family from Northern Pakistan. In addition to expanding the known mutational repertoire of *PDE6B*-linked RP, our findings suggest that exome sequencing coupled with autozygosity mapping remains a useful diagnostic tool in genetic investigations involving consanguineous families, thus warranting further studies in the country to explore the full genetic spectrum of IRDs and other Mendelian disorders. Finally, our results may be helpful for the respective family in seeking disease management of their patients as well as for carrier testing and genetic counselling.

## Supplementary Information

**Additional file 1: Supplementary Table S1.** List of interactions of wild type and mutated *PDE6B* with *PDE6A*.

## Data Availability

The datasets generated and/or analyzed during the current study are available in the ClinVar database, [Accession SCV002540278].

## Abbreviations

arRP: Autosomal Recessive Retinitis Pigmentosa
adRP: Autosomal Dominant Retinitis Pigmentosa
ACMG: The American College of Medical Genetics and Genomics
CADD: Combined Annotation Dependent Depletion
DNA: Deoxyribonucleic acid
ExAC: Exome aggregation consortium
HGMD: Human Gene Mutation Database
gnomAD: Genome aggregation database
OCT: Optical coherence tomography
SIFT: Sorting Intolerant from Tolerant
