# Supplementary material for: A novel homozygous missense substitution p.Thr313Ile in the PDE6B gene underlies autosomal recessive retinitis pigmentosa in a consanguineous Pakistani family
Source: BMC Ophthalmol. 2023 Mar 23;23:116. doi: 10.1186/s12886-023-02845-0 (PMC10035148; doi:10.1186/s12886-023-02845-0)
Supplement: Supplementary file 1 — Additional file 1: Supplementary Table S1. List of interactions of wild type and mutated PDE6B with PDE6A. [file 12886_2023_2845_MOESM1_ESM.docx]

Supplementary Table S1: List of interactions of wild type and mutated *PDE6B* with *PDE6A*.

| **Wild Type Interactions** | | | **Mutated Type Interactions** | | |
| --- | --- | --- | --- | --- | --- |
| **Residue-of-****PDE6A** | **Residue-of**  **PDE6B** | **Bond-Length** | **Residue-of**  **PDE6A** | **Residue-of**  **PDE6B** | **Bond-Length** |
| Glu-298 | Lys-402 | 2 | Glu-287 | Ser-343 | 2 |
| Glu-287 | Arg-348 | 1.8,1.9 | Glu-287 | Arg-348 | 1.9,1.9,2.4 |
| Ser-246 | Lys-112 | 1.8,1.7 | Asp-452 | Arg323 | 1.8,2.0 |
| Asn-433 | Thr-155 | 3.2 | Asp-452 | Arg-273 | 1.8,1.9 |
| Asn-433 | Asn-152 | 1.9 | Tyr-456 | Arg-274 | 2.0,2.1,2.6,1.9 |
| Arg-629 | Glu-6 | 1.7 | Asp-461 | Arg-274 | 1.8 |
| Lys-676 | Glu-17 | 2 | Lys-459 | Arg-274 | 2.5 |
| Lys-620 | Asn-164 | 1.7 | Arg-446 | Glu-352 | 1.9,2.2 |
| Glu-633 | Arg-165 | 2.0,1.9 | Glu-633 | Arg-165 | 1.8,2.1 |
| Lys-422 | Lys-339 | 2.6 | Lys-620 | Asn-164 | 1.7 |
| Arg-446 | Glu-352 | 2.1,2.0 | Arg-629 | Glu-6 | 2.1,1.9,1.9 |
| Arg-604 | Asp-171 | 2 | Arg-604 | Asp171 | 1.8,2.0 |
| Arg-554 | Asp-171 | 1.9,2.0 | Arg-554 | Asp171 | 1.9,2.2 |
| Lys-555 | Asp-180 | 1.9,1.8 | Lys-555 | Tyr-175 | 1.9,-1.7 |
| Lys-555 | Glu-183 | 2 | Lys-555 | Asp-180 | 1.9 |
| Asp-452 | Arg-323 | 1.9,2.2 | Lys-555 | Glu-183 | 1.8 |
| Asp-452 | Arg-273 | 1.8,1.8 |  | | |
| Tyr-456 | Arg-274 | 2.0,2.0 |  |  |  |
| His-457 | Gly-271 | 3.1 |  |  |  |
| His-457 | Lys-270 | 3.2 |  |  |  |
